# The social learning and development of intra- and inter-ethnic sharing norms in the Congo Basin

**DOI:** 10.1371/journal.pone.0340388

**Published:** 2026-01-28

**Authors:** Sheina Lew-Levy, Luke Maurits, Adam H. Boyette, Kate Ellis-Davies, Daniel Haun, Wilson Vieira, Ardain Dzabatou, Bienvenue Mbongo, Francy Kiabiya Ntamboudila, Roger Ndenguele, Harriet Over, Bailey R. House, Sarah Pope-Caldwell

**Affiliations:** 1 Department of Psychology, Durham University, Durham, United Kingdom; 2 Department of Comparative Cultural Psychology, Max Planck Institute for Evolutionary Anthropology, Leipzig, Germany; 3 Department of Human Behavior, Ecology, and Culture, Max Planck Institute for Evolutionary Anthropology, Leipzig, Germany; 4 Department of Psychology, Swansea University, Swansea, United Kingdom; 5 Leipzig Research Centre for Early Child Development, Leipzig University, Leipzig, Germany; 6 Faculté des Lettres, Arts, et Sciences Humaines, Marien Ngouabi University, Brazzaville, Republic of the Congo; 7 Department of Psychology, Georgia State University, Atlanta, Georgia, United States of America; 8 Association des Jeunes pour l’Education a la Sauvegarde des Eléphants au Congo, Brazzaville, Republic of the Congo; 9 Department of Psychology, University of York, York, United Kingdom; University of Michigan, UNITED STATES OF AMERICA

## Abstract

Compared to other species, the extent of human cooperation is unparalleled. Such cooperation is coordinated between community members via social norms. Developmental research has demonstrated that very young children are sensitive to social norms, and that social norms are internalized by middle childhood. Most research on social norm acquisition has focused on norms that modulate intra-group cooperation. Yet around the world, multi-ethnic communities also cooperate, and this cooperation is often shaped by distinct inter-group social norms. In the present study, we investigated whether intra-ethnic and inter-ethnic social norm acquisition follows the same, or distinct, developmental trajectories. Specifically, we worked with BaYaka foragers and Bandongo fisher-farmers who inhabit multi-ethnic villages in the Republic of the Congo. In these villages, inter-ethnic cooperation is regulated by sharing norms. Based on our ethnographic knowledge of the participating communities, we predicted that children’s intra-ethnic sharing choices would match those of adults at an earlier age than their inter-ethnic sharing choices. To test this prediction, children (5–17 years) and adults (17 + years) participated in a modified Dictator Game to investigate the developmental trajectories of children’s intra- and inter-ethnic sharing choices. Contrary to our prediction, both intra- and inter-ethnic sharing norms were acquired in middle childhood. Interviews with adult participants suggested that intra- and inter-ethnic sharing norms are acquired from multiple sources, including parents and peers. Further, Bandongo adults primarily reported learning sharing norms via Instruction, whereas BaYaka adults primarily reported learning via Observation/Imitation. These cross-cultural differences may reflect variation in norm complexity. Together, these findings suggest that when social contexts regularly expose children to out-group collaboration, inter-ethnic norms are acquired at similar timelines to intra-ethnic ones, as part of children’s broader cooperative repertoire.

## Introduction

Cooperation is central to the success of our species [1,2]. Humans cooperate across a range of daily activities including subsistence [3], food sharing [4,5], childcare [6,7], and knowledge transmission [8,9]. Compared to other primates, human cooperation is unique in the extent to which it varies across communities [10,11], and in that we cooperate with many unrelated and even unknown individuals [12,13]. Social norms, or “mutual agreements or commitments about the way that individuals ought to behave in certain situations” [8], ensure that cooperation is coordinated between community members [14–16]. In turn, ethnic markers such as language, behaviours, and styles of dress help community members identify each other, hence facilitating in-group cooperation [14,17].

Most research into the development of social norms has focused on intra-group cooperation. These studies have demonstrated that children are sensitive to social norms by the age of three [18], that children internalize social norms by middle childhood [19], and that children as young as three preferentially cooperate with in-group members [20]. Yet, around the world, many individuals live in multi-ethnic communities characterized by inter-group cooperation [21–24]. Inter-group cooperation may help communities manage risks associated with resource shortfalls and provide access to nonlocally available resources [25–27]. In such communities, individuals not only have social norms for cooperating *within* their ethnic groups, but *between* them.

In the present paper, we aim to investigate the development of intra- and inter-ethnic social norms in multi-ethnic villages inhabited by BaYaka foragers and Bandongo fisher-farmers in the Republic of the Congo. These communities regularly engage in inter-ethnic cooperation regulated by sharing norms. In this study, we provide the first descriptive account of the timing and mechanisms by which BaYaka and Bandongo learn to share with out-group members via interviews with child and adult participants. We also use a modified Dictator Game to investigate the developmental trajectories of children’s intra- and inter-ethnic sharing choices. Considerable research has been done to develop experimental tasks measuring sharing across a wide range of ages and communities, providing a firm methodological footing for this experimental paradigm. In what follows, we summarize findings from previous studies on the development of intra-group social norms and inter-group biases. We then describe the context in which the present study took place.

### Developing social norms

Prosocial behaviour emerges in infancy [28] and increases in both frequency and sophistication between early childhood and adolescence [29,30]. Children as young as three rapidly infer the presence of norms, protesting the incorrect usage of an object after having seen it used ‘correctly’ by an adult model only once [18]. By age five, children spontaneously generate their own norms in novel collaborative games [31], and protest norm violations that would benefit them in a competitive game [18]. German and American three- to five-year-old children show a clear willingness to conform to the behaviour of others [32–34].

Societal variation in prosocial behaviour begins to increase around 7–10 years of age. For example, studies have found increasing societal differences in generosity in a binary Dictator Game [19,29,35] and advantageous inequity in the Inequity Aversion Game [36,37]. Children in middle childhood also modify their prosocial behaviour in response to normative information. For example, 6- to 9-year-old German children were more likely to share in a binary Dictator Game when their knowledge of local norms was primed (i.e., when they were told they could “share like they think they ought to share”) relative to when their own preferences were primed (i.e., when they were told that they could “share as they wished”) [38]. This suggests that these children held knowledge of a norm specifying that they ‘ought to share.’ Researchers have also found that 6- to 10-year-old German and British children’s prosocial behaviour in the binary Dictator Game becomes increasingly influenced by norms as they age [39,40]. Taken together, these findings suggest that by middle childhood, children become increasingly sensitive to, and likely to conform to, community-specific norms. However, how social learning contributes to variation in the timing of norm acquisition has been infrequently explored.

### Developing in-group preferences

Inter-group bias appears early in development. American five-year-olds show consistent preferences for members of their own age group [41], gender [42,43] and language group [44]. Sensitivity to group membership cues also shape children’s social learning. Buttelmann and colleagues [45] showed that 14-month-old German children were more likely to imitate the actions of speakers of their own language than speakers of a different language. In comparison, when presented with an action performed by out-group members, 5-year-olds were more likely to perform a contrasting action than the one observed [46].

Children are more likely to act cooperatively with in-group members [44,47–50]. For example, 2.5-year-old American children are more likely to share toys with native language speakers [20]. Children from western cultural contexts are also more likely to help and share with members of their own ethnic groups [48,51–53]. Even arbitrary or transient in-groups result in increased prosocial behaviour. American preschoolers preferentially allocate resources to randomly classified in-group members marked by armband and sticker colours [54]. Yet, norms may play an important role in regulating inter-group sharing. When equitable, Swiss second-graders adhere to suggested in-group and out-group sharing norms [55]. Inter-group cooperation may be enhanced in communities where strong social norms regulate inter-ethnic interactions [56].

### Learning about social norms and group membership

Ethnographic research suggests that several social learning mechanisms contribute to children’s growing awareness of community-specific social norms, including sharing norms [57,58]. Parents play an active role in teaching children to share in early life. For example, when Kalahari San eight-month-olds give objects to others, parents actively encourage them [59]. In early childhood, Indian Nayaka parents send children to distribute plates of food to other households [60]. When children refuse to share, Central African Aka caregivers may withhold food, gossip about them, hit them, or insult them [61]. In some Chinese schools, teachers actively provide instruction related to fairness through disciplinary and motivational interventions, peer comparisons, and moral comments [62]. Children also learn sharing norms in child-only groups. For example, a Congolese BaYaka child may carefully dole out tiny portions of food during play, sending these portions to other children in a manner emulating adult sharing [63]. In peer groups, Israeli children participate in ritualized sharing of candy and other treats [64]. While foraging, Tanzanian Hadza children share food with their peers, and abstain from consuming food so that they can share them with their caregivers upon return to camp [65,66].

Social learning also plays an important role in children’s acquisition of beliefs about in- and out-group membership and their attitudes towards them [67–70]. In terms of the acquisition of stereotypical beliefs, a large body of research has shown that children are exposed to cultural stereotypes in conversation with parents as well as through the broader culture [70]. In terms of the acquisition of inter-group attitudes, a comprehensive meta-analysis of more than 45,000 parent-child dyads from predominantly western cultural contexts reported a moderate and positive relationship between the attitudes of children and those of their parents [71]. Supporting this view, Skinner and colleagues [72] demonstrated that observing negative non-verbal behaviour towards a stranger was sufficient to lead American children to hold a negative attitude towards that person and towards that person’s friends. Interactions between children may also shape their out-group attitudes; Peruvian Matsigenka children adopted the norms of their Mestizo neighbours through sustained inter-ethnic interactions, primarily at school [73]. However, we know of no ethnographic studies that have described how children learn inter-ethnic social norms, despite their importance to inter-group cooperation.

### Ethnographic setting

Our study explores the development of intra- and inter-ethnic sharing norms among BaYaka and Bandongo inhabiting two villages along the Motaba river in the Likouala Department of the Republic of the Congo [74]. Bandongo are primarily fisher-farmers who also participate in hunting and trapping [75]. BaYaka are foragers who primarily collect honey, wild yams, mushroom, fish, wild game, and other forest products, supplemented by cultigens from low-intensity gardens [76,77]. BaYaka and Bandongo primarily use linguistic (Yaka, Bondongo/Lingala) and behavioural markers to distinguish between their communities. BaYaka view Bandongo as accumulators of wealth, hierarchical, and as claimants of forest areas as their own [78]. Bandongo identify BaYaka based on their sociability, lack of food reserves (reflecting their immediate-return economy), and their knowledge of the forest [78].

BaYaka intra-group sharing norms are organized around generalized giving [79,80]. Consistent with their strong egalitarian ethos, having a resource is understood by BaYaka as having an obligation to share it, and an expectation that others have the right to demand it [81]. Food sharing norms are formalized into specific food taboos about how hunters allocate their kills, based on gender and specific roles during the hunt. Portions of cooked meals are typically shared according to kinship, residential proximity, and need [82]. Sharing of most resources is unconditional; failing to share is not only viewed as inviting social discord into the community, but also as angering the forest, thus threatening the future availability of resources [83].

Among the many ethnic groups of farming and fishing peoples in the region, including Bandongo, sharing norms reflect the cultural values of family communalism and status hierarchy [84]. Resource production and consumption typically occurs along the patriline. Sharing within households is governed by gender and age. Resources are considered the private property of the family. Within extended families, sharing is governed by norms of obligation to specific relatives (e.g., elders, in-laws). Between families, resources are exchanged via barter or sale, and sharing is largely constrained to community-wide events, such as funerals and rites of passage. Generally, people keep track of debts either formally or informally. Individuals or families deemed as having much more than others are accused of using witchcraft. Such threats help avoid disproportionate accumulation.

Sharing between BaYaka and Bandongo occurs in the context of economic exchange relationships, typically institutionalized through fictive kinship [85]. BaYaka men are often hired as shotgun hunters for Bandongo. In these contexts, BaYaka receive the hunter’s portion of meat, including the head, the tail, and the guts. They also receive a pre-agreed gift, such as a headlamp or clothing [86,87]. BaYaka women routinely help build Bandongo houses, in exchange for palm wine and a manioc dish called *jabuka* (Yaka) or *pondu* (Lingala)*.* BaYaka women collect caterpillars which they exchange for baby clothes and bassinets. Both BaYaka men and women contribute to farming labour in exchange for cultigens such as manioc, plantains, and corn. Finally, BaYaka and Bandongo inherit overlapping sections of the forest. Forest resources are jointly managed through harvesting and sharing rules. It is important to note that these sharing norms are not without contention. Conflicts can arise when one party considers resources to be unfairly shared, when debts have not been paid, or when an area of the forest no longer produces sufficient resources. In such cases, institutions such as council meetings and *nganga* (traditional doctor) healing ceremonies help mitigate potential inter-ethnic conflict [88].

BaYaka children begin to learn intra-ethnic sharing norms in infancy, and socialization of sharing continues throughout childhood [89]. For example, children say their mothers taught them to share by showing them how to allocate portions of the evening meal [61]. Women then call upon children to distribute these plates to specific members of the community [89]. Sharing norms are also likely reinforced though the practice of demand sharing, which means that anyone has a right to ask another for a portion of a resource, and they are obligated to abide. Enforcement of sharing norms is indirect, and typically individual transgressions are not specifically sanctioned. Rather, adults will refer to improper sharing as the cause of failures to catch game on hunts, or they will denounce selfishness in a general way during public speeches (*mosambo*) or through satirical reenactments of selfish or improper behavior (*moadjo*) [88,90]. While less is known about how Bandongo learn sharing norms, children, especially girls, are involved in food preparation from early in life and have the same opportunities BaYaka children do to observe their parents’ sharing practices. As noted above, accusations of witchcraft are one serious form of public norm enforcement that children would witness.

How or when children learn inter-ethnic sharing norms is less understood. In early and middle childhood, BaYaka and Bandongo children may accompany their parents and observe while they participate in inter-ethnic labour or exchanges. However, it is not until early adolescence that inter-ethnic sharing becomes common. Bandongo adolescents hire BaYaka peers to go hunting to raise sufficient funds for the upcoming school year, often living in forest camps together for extended periods of time. BaYaka and Bandongo adolescent girls accompany their mothers to Bandongo fields. Bandongo pre-adolescent and adolescent children are also sometimes sent to collect debts from BaYaka for their parents. Such experiences provide extensive opportunities for adolescents to interact with out-group members, learn about each other’s sharing norms, observe their parents barter and trade, and participate in exchanges themselves. Explicit teaching of inter-ethnic sharing norms may occur during *mosambo* and *moadjo* among BaYaka, when adults counsel adolescents in how to behave, and reprimand them when they have violated an inter-ethnic norm [91,90]. Among the Bandongo, parents actively counsel their children regarding inter-ethnic sharing norms (Kandza, personal communication). Both BaYaka and Bandongo children also participate in village council meetings, where inter-ethnic norms are often discussed, and violations are resolved. The village crier (*mopandji sango*), who walks through both BaYaka and Bandongo neighbourhood in the evening sharing news of the day’s activities as well as any decisions taken by the village council, often reminds community members to cooperate by respecting intra- and inter-ethnic sharing norms (Kandza, personal communication).

### The present study

As outlined, previous experimental research has demonstrated that children are sensitive to social norms and group membership in early childhood. By middle childhood, children have internalized community-specific social norms, leading to cross-cultural variation in behaviour. Social learning research further suggests that children develop social norms and inter-group attitudes from parents and other children via teaching, observation, and practice during play and work activities. Here, we add to this body of research by investigating the social learning and developmental trajectories of intra- and inter-ethnic sharing norms in a multi-ethnic community in the Republic of the Congo where inter-ethnic cooperation is common. Specifically, we aim to describe how, when, and from whom BaYaka and Bandongo learn inter-ethnic sharing norms via interviews with children and adults. We also aim to investigate how adult-like intra- and inter-ethnic sharing norms develop using a modified Dictator Game. Our ethnographic work suggests that BaYaka and Bandongo children learn intra-ethnic sharing norms in early childhood, whereas learning inter-ethnic sharing norms may occur more intensively in adolescence. We thus predict that children’s intra-ethnic sharing choices in the Dictator Game will match those of adults at an earlier age than their inter-ethnic sharing choices.

## Methods

Data were collected in two multi-ethnic villages of approximately 700–800 BaYaka and Bandongo inhabitants each with similar demographic profiles (though on-site population varies based on seasonal mobility), subsistence strategies, access to markets, and distances from urban centres [92]. In our previous experimental and ethnographic work in these two villages, we have observed intra- and inter-ethnic sharing practices commensurate with regional social norms. Experiments and short post-experiment interviews were conducted between August 16^th^ and October 1^st^ 2023. Additional interview data were collected between March 5^th^ and April 6^th^ 2025. All consent, interview, and experiment scripts were forward and back-translated into Lingala for Bandongo participants and Yaka for BaYaka participants. Note that, because BaYaka ages are not usually documented, we used age estimates (in years) based on birth order (see [93] for details) when available, and estimates by local research assistants otherwise. Age in years is typically known for Bandongo. The Registered Report Protocol for this study is published as [94]. Deviations from this protocol are outlined in the supplemental materials.

### Approvals

Ethical approval was obtained from the Durham University Psychology ethics committee and from the Max Planck Group ethics committee. In-country permission was obtained from the Congolese National Institute for Research in Social and Human Sciences. Consent was obtained in accordance with local cultural norms as established in previous field seasons. Specifically, community consent was obtained during village meetings hosted by Bandongo and BaYaka leaders. During these meetings, we described the goals of the study. We emphasized that participation is not mandatory, and that individuals can withdraw from the study at any time without penalty. We answered any questions the community had. The community decided by consensus that we could conduct research in the villages.

Following community consent, we sought individual verbal consent from adults. We reiterated the goals of the research, the research procedure, the gifts that would be received irrespective of participation, and that participants could withdraw at any time. We answered any questions, and where applicable, asked for parent/guardian consent for child participation.

Adult and child assent was obtained again in the testing room immediately prior to administering the experiment using a translated and back-translated assent script. In addition to verbal assent, we paid careful attention to children’s shyness or apparent discomfort. If, prior to the start of the experiment, or at any point throughout it, a child verbally or non-verbally signaled that they did not wish to participate, we stopped the experiment and moved on to the next participant. Participants received culturally appropriate gifts commensurate with local sharing norms and the time that they spent working with us.

### Interview

Interviews were conducted with 66 adults (58% Women; 47% BaYaka; 52% Village 1). For both intra-ethnic and inter-ethnic sharing, we asked participants to free-list the cultural models (as categories, e.g., mother, friend) from whom they learned to share [84]. These were recoded to facilitate comparison (e.g., ‘older peer’, ‘younger peer’, ‘peer’, ‘friend’ were recoded as ‘peer’). We asked participants to identify the stage of childhood during which they began to learn to share following local cultural understandings of child development which roughly map on to early childhood (*mona mosoni*/*mwana moke*), middle childhood (*mona akoka/mwana ya mokolo*), and adolescence (*mosonde* (boys), *ngondo* (girls)/*elenge*). Finally, participants were asked to describe how they learned sharing norms within and across ethnicity. These latter descriptions were independently recoded into teaching and learning types adapted from Hewlett and Roulette [95] by SLL and SPC (see Table 1 for definitions). If the participant reported more than one learning mechanism, we only coded the first. Intercoder reliability was high (96% agreement; kappa = 0.93); all disagreements were resolved by discussion.

**Table 1:** Description of teaching and learning categories used to code the interview responses, adapted from Hewlett and Roulette [95].

Type	Definition
**Demonstration**	Other shows the learner how to share.
**Task assignment**	Other tasks the learner to share.
**Instruction**	Other explains, tells, or gives advice on how sharing should or should not be undertaken, either *in situ* or through public speaking (e.g., storytelling, preaching)
**Observation/Imitation**	The learner observes sharing and/or imitates the caregiver’s sharing, including in the context of reciprocity (i.e., someone shares with the learner, and the learner shares back)
**Play**	The learner emulates sharing during play.
**Self**	The learner learns to share independently from others.

A short interview was conducted with all participating BaYaka and Bandongo children (5–16 years) immediately after the Dictator Game (see below). In total, 119 children (M_age_ = 9.74, SD = 2.82; 46% Girls; 51% BaYaka; 45% Village 1) were asked to report whether they knew how to share within and across ethnicity. We chose interview questions with a binary response (yes/no) because these could be communicated non-verbally (e.g., by shaking one’s head), thus helping overcome child participant’s shyness common in experimental contexts [84]. Four participants declined to respond to, or did not know the answer to, *both* intra- and inter-ethnic sharing questions. After excluding these participants, our final sample consisted of 115 participants.

We conducted open-ended (i.e., ethnographic) group interviews with adults to understand changes in inter-ethnic sharing norms across generations, and whether mobility shapes inter-ethnic sharing norms. Specifically, we conducted 13 same-gender, same-ethnicity, same-village focus groups of 5 participants each (S1 Table). Questions included: (1) How did the ancestors (*bakoko*) share across ethnicity? (2) How do you share across ethnicity? (3) Are children today learning to share differently than in the past? (4) How did BaYaka and Bandongo interact in the past? (5) How have these interactions changed? (6) How do you resolve a conflict related to inter-ethnic sharing today (e.g., debt)? (7) How were these conflicts resolved in the past? (8) Do BaYaka/Bandongo who live in the forest full-time or almost full-time have different intra- and inter-ethnic sharing norms than those who spend more time in the village? Similarly, we conducted open-ended (i.e., ethnographic) group interviews with 12 same-gender, same-ethnicity, same-village focus groups of 5 older child/adolescent participants each. Questions included: (1) From whom did you learn intra-ethnic sharing norms? (2) From whom did you learn inter-ethnic sharing norms? (3) How do you share within ethnicity? (4) How do you share across ethnicity? For both adults and children, group interviews were preferred because participants were more comfortable elaborating on their ideas in the company of their peers.

### Dictator Game

#### Participants and exclusions.

We recruited 122 children (5–16 years) and 83 adults (17 + years) for a total of 205 participants (M_age_ = 20.71, 47.3% girls/women, 50.7% BaYaka). We excluded participants from our final sample if (1) they did not complete all relevant elements of the study (i.e., opted out prior to testing) (n = 5), (2) they were excessively shy, anxious, or uncomfortable during testing (n = 5), (3) they did not pass all comprehension checks embedded in the experiment (n = 11) or (4) if a substantive experimenter error occurred (n = 5). Exclusions were coded by a research assistant who reviewed all video data, then checked by another research assistant as part of the intercoder reliability checks. Comprehension checks were designed to require minimal verbal communication to help overcome participant shyness. We opted for questions that could be answered via pointing, or in the case of counting, by holding up one or two fingers. All participants were given three chances to pass the comprehension tests. If a participant failed any comprehension test thrice, the experimental program immediately ended the experiment, and the participant was excluded from the study. After exclusions, our final sample consisted of 179 participants.

#### Overview of experiment.

Participants played a binary choice Dictator Game. The Dictator Game has been successfully administered with neighbouring Aka forager children in the Central African Republic [19]. We designed aspects of this Dictator Game to be culturally sensitive and salient. In the classic Dictator Game, participants are presented with tokens that represent some unknown quantity. Participants exchange these tokens for rewards. To accommodate the immediate-return economy of BaYaka participants, we adjusted this Dictator Game to include the rewards (candy) as the currency in the game [35]. During the experiment, participants made a series of choices between two predetermined payoff distributions. Specifically, participants could choose to equally distribute two candies between themselves and another person (GIVE) or keep both candies for themselves (KEEP). This occurred over the course of two trials corresponding to two conditions in which the potential recipient was described as either the same ethnicity or a different ethnicity.

#### Testing context and materials.

In each village, testing occurred in a quiet room in the research house. One BaYaka and one Bandongo research assistant was trained to administer the experiment to co-ethnic participants. The apparatus consisted of two laminated paper trays 3.5 x 8.5 inches each with a yellow and purple circle on either end. Each tray represented a payoff distribution (GIVE or KEEP). Candies placed on the yellow circle facing participants were for participants, and candies placed on the purple circle further from participants were for the recipient. Meeples, small humanoid figurines, were used to represent the participant and recipients, such that co-ethnic recipients were the same colour (blue or red) as the participant and vice versa. Participants and recipients remained anonymous to each other. Research assistants were guided through the experiment using the Open Data Kit application on a tablet [96]. Participant responses and choices were recorded within the application. The location of the payoff distribution (left or right tray from the perspective of the participant), the meeple colour assigned to each ethnicity (blue or red), and the order of conditions (same ethnicity or different ethnicity recipient) were randomized automatically within the application. We opted to use these randomly-assigned visual markers of ethnicity because, as previously mentioned, BaYaka and Bandongo primarily distinguish each other through linguistic and behavioural markers, which do not straightforwardly translate to this experimental paradigm. Randomly-assigned colours have been used successfully as in- and out-group markers in previous experiments in the post-industrialized west [54], and are also used as part of team uniforms at the field site during inter-village football games. Thus, we expected that assigning meeple colours to ethnicity would be well understood in this context; that fewer than 3% of participants failed this comprehension check suggests our expectation was met. Testing was video recorded in case of equipment malfunction and to conduct intercoder reliability. We calculated intercoder reliability for 25% of experiments. Reliability was high (Trial 1: 100% agreement; kappa = 1.0; Trial 2: 98% agreement; kappa = 0.96; To Exclude: 98% agreement; kappa = 0.85).

#### Procedure.

**Fig 1:**
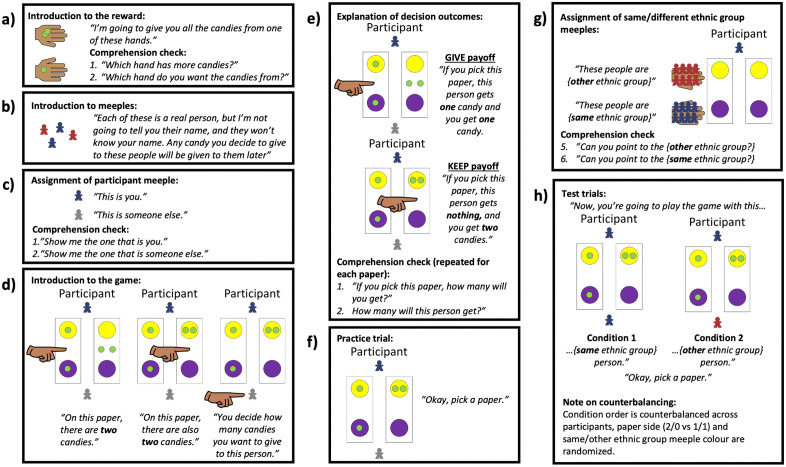
Dictator Game procedural details. (a) introduction to reward; (b) introduction to meeples and emphasis on their representation of real people; (c) assignment of participant’s meeple colour; (d) introduction to game play; (e) explanation of decision outcomes; (f) practice trial; (g) assignment of same/different ethnic group meeples; (h) test trials.

Full procedural details are outlined in Fig 1. The testing procedure had 9 steps:

1. Introduction to the reward: The experimenter hands the participant a cup. The experimenter places two candies in one hand, and one candy in the other. The experimenter holds out their hands to show the candies to the participant. The experimenter says: “I’m going to give you all the candies from one of these hands.”

Comprehension check: The experimenter asks the participant to point to the hand with more candies. The experimenter then asks the participant to choose a hand from which to collect the candies. Participants **pass** the comprehension test if they correctly identify the hand with more candies.

Distribution of reward: The experimenter places the participant’s candies in their cup.

1. Introduction to meeples: The experimenter picks up a handful of meeples and shows them to the participant. The experimenter says: “Each of these is a real person, but I’m not going to tell you their name, and they won’t know your name. Any candy you decide to give these people will be given to them later.”
2. Assignment of participant meeple: The experimenter shows the participant a red OR blue meeple (counterbalanced) and a says: “This is you.” The experimenter shows the participant a grey meeple and says: “This is someone else.”

Comprehension check: The experimenter asks the participant to point to the meeple that represents them. The experimenter then asks the participant to point to the meeple that represents someone else. Participants **pass** the comprehension test if they correctly identify the meeples.

1. Introduction to the game: The experimenter pulls out the trays, and places them such that the yellow circles face the participant. The experimenter places the participant’s meeple in front of the yellow circle, and the grey meeple across from the participant’s meeple, in front of the purple circles. The experimenter places two candies on the center of each tray. The experimenter points to the left OR right tray (counterbalanced) and says: “On this paper, there are two candies”. The experimenter places one candy in the yellow circle, and one candy in the purple circle (GIVE payoff). The experimenter points to the second tray and says: “On this paper, there are also two candies.” The experimenter places two candies in the yellow circle, and none in the purple circle (KEEP payoff). The experimenter points to the grey meeple and says: “You decide how many candies you want to give to this person.”
2. Explanation of decision outcomes: The experimenter points to the GIVE tray and says: “If you pick this paper, this person gets one candy, and you get one candy.” The experimenter then points to the KEEP tray and says: “If you pick this paper, this person gets nothing, and you get two candies.”

Comprehension check: The experimenter points to each of the GIVE and KEEP trays and asks the participant how many candies they will get, and how many candies the recipient will get, if they pick each tray. Participants **pass** if they correctly identify the number of candies they will keep and give for each tray.

1. Practice trial: The experimenter says: “Okay, pick a paper.” The experimenter records the participant’s response.

Distribution of reward: The experimenter places the grey meeple in a cup, and places one or no candies (depending on the participant’s choice) in that cup. The experimenter places the participant’s candies in their cup.

1. Assignment of same/different ethnic group meeples: The experimenter picks up a handful of red meeples in one hand, and blue meeples in another. The experimenter shows the participant the red OR blue meeples (matching the participant’s meeple colour) and says: “These people are BaYaka OR Bandongo (matching the participant’s ethnicity).” The experimenter shows the participant the other meeples and says: “These people are Bandongo OR BaYaka (contrasting the participant’s meeple colour and ethnicity).”

Comprehension check: The experimenter asks the participant to point to the meeples of the participant’s same ethnic group. The experimenter asks the participant to point to the meeples of the other ethnic group. Participants **pass** the comprehension test if they correctly identify the ethnicity of the meeples.

1. Test trials: The experimenter sets up the trays as in step 4. The experimenter then presents the two conditions, the order of which are counterbalanced across participants, with all participants participating in both conditions. The experimenter places a same-ethnicity meeple (condition 1) or an other-ethnicity meeple (condition 2) in front of the purple circles. The experimenter says: “Now, you’re going to play the game with this BaYaka OR Bandongo person (same ethnicity for condition 1, other ethnicity for condition 2).” The experimenter says: “Okay, pick a paper.” The experimenter records the participant’s response. The experimenter places the recipient meeple in a cup, and places one or no candies (depending on the participant’s choice) in that cup. The experimenter places the participant’s candies in their cup.
2. Post-interview questions & thank you: We conducted the short post-interview questions with children, and thanked all for their participation.

### Data analysis

All statistical analyses were conducted in R [97].

#### Qualitative interview analysis.

Open-ended interviews were video recorded, then translated and transcribed from Lingala/Yaka to French by trained research assistants, and from French to English by SLL, who is fluently bilingual. Salient qualitative trends were identified and summarized within an ethnographic framework [98]. Specifically, SLL read through the transcripts, attending to differences and similarities between BaYaka and Bandongo accounts of inter-ethnic cooperation, sharing norms, and generational changes within these. She noted any observations that were recurrent, surprising, or contradictory. These observations guided later transcript readings in which emerging patterns were refined, and illustrative quotes were extracted. SPC then read through the transcripts to ensure that our interpretation accurately reflected the patterns and nuances of participants’ accounts.

#### Interview descriptive & exploratory statistics.

For *whom* adult participants learned intra- and inter-ethnic sharing from, we report the frequency and Smith’s Salience [99] (i.e., the average percentile rank of an item across all lists) for each category of cultural model, separated by participant ethnicity and intra-/inter-ethnic sharing. A salience nearing 1 would reflect the fact that nearly all participants named the cultural model near the top of their list, whereas a salience closer to 0 would reflect the fact that few participants named the cultural model, and/or most placed them at the end of their list. Salience scores were calculated in AnthroTools [100].

For *how* adult participants learned intra- and inter-ethnic sharing, we report the total number and percent of adult participants who learned intra- and inter-ethnic sharing via each teaching and learning type, separated by participant ethnicity.

To understand reported age of sharing knowledge acquisition among adults, we fit a statistical model to interview responses. Specifically, in a multinomial regression, we predicted the reported age of sharing acquisition using gender, condition (i.e., intra- or inter-ethnic sharing), and ethnicity as main effects, and a two-way interaction between condition and ethnicity. To understand age of sharing knowledge acquisition among the children, we analyzed the post-interview yes/no responses to knowledge of intra- and inter-ethnic sharing. Specifically, in a logistic regression, we predicted knowledge of sharing using age (*z*-score standardized), gender, condition, and ethnicity as main effects, and a three-way interaction between condition, age, and ethnicity. As data for both models involved repeated observations for individuals, we also included a random effect for participant. Both models were fit in BRMS [101] on 4 chains of 10,000 iterations each. We specified weakly informative priors in both cases. Importantly, we use these models *not* as statistical tests, but as a way to visualize general trends in the data adjusting for variation in gender, age, and ethnicity in our sample.

#### Modelling experimental data.

Experimental data were analyzed using a Bayesian modelling approach [100], with estimation performed via Hamiltonian MCMC using Stan [88] and Rstan [101]. The developmental trajectories of sharing norms were modelled using logistic curves, which transition smoothly between an initial infant and final adult probability for sharing. For each of the two societies, two curves were fit to the data, one representing the development of intra-ethnic sharing norms and one representing the development of inter-ethnic sharing norms, totalling four curves for the study. A single initial infant probability was estimated across all four developmental trajectories, encoding the idea that the behaviour of infants, prior to socialization, is not expected to vary across society or condition. The shapes of the four curves were estimated independently using data from the appropriate society and condition, with four separate final probabilities allowed.

Each curve’s shape was determined by two parameters, one of which is directly interpretable as the age at which individuals in that society are halfway between infancy and adulthood in terms of the norm acquisition, in the sense that their sharing rates are equal to the mean of the initial sharing rate and their society’s adult sharing rates. The second parameter dictates how gradual or rapid the transition is. In mathematical terms, focusing on a single society and a single condition (i.e., intra- or inter-ethnic sharing), and denoting a participant’s sharing choice by y, their age by x, the universal “infant” sharing probability by p_infant_, the (society-specific, condition-specific) adult sharing probability by p_adult,_ and the two (society-specific, condition-specific) parameters by a and b, then the full model specification including priors is:


$$p_{\text{infant}}\;\sim \;\text{Beta}\;(\text{1}.\text{5},\;\text{1}.\text{5})$$

$$p_{\text{adult}}\;\sim \;\text{Beta}\;(\text{0}.\text{75},\;\text{0}.\text{75}),$$

$$\left[\begin{array}{c} a\\ b \end{array}]\;\sim \;N\;([\begin{array}{c} \text{1}\text{0}\\ \text{0}.\text{2} \end{array}],[\begin{array}{cc} \text{1}\text{5} & \text{0}.\text{7}\text{5}\\ \text{0}.\text{7}\text{5} & \text{0}.\text{2} \end{array}]\right)$$

$$y\;\sim \;\text{Bernoulli}\;\left(p_{\text{infant}}+\frac{p_{\text{adult}}-p_{\text{infant}}}{\text{1}+\text{exp}(-b(x-a))}\right)$$

For all four curves, a multivariate normal prior distribution, truncated to allow only positive values, was placed on the two parameters, with a positive correlation such that curves where development ‘starts later’ are also more likely to be curves where development ‘happens quicker’ to avoid development continuing into adulthood. In addition, and deviating from our Registered Report Protocol [93], we included participant gender in the models to account for unanticipated imbalance in the sample distribution: the parameters a and b above were replaced by a + za_g_ and b + zb_g_, with z equal to ± 0.5 according to gender, so that the multivariate normal prior above is specified for the average of the two gender-specific effects. Similarly, p_adult_ was replaced by logit^-1^(logit(p_adult_) + zp_g_). The a_g,_ b_g_ and p_g_ gender effects were all given N(0, 0.75) priors.

For any one of the four development curves, we can calculate a “completion age” corresponding to the age when 95% of the change between the infant and adult sharing probabilities has taken place. This has happened when exp(-b_ij_(x – a_ij_)) = 0.05, i.e., at age x ≈ a + 3/b. Fitting the model to data provides a joint posterior distribution for each of the a and b parameters, which translates to posterior distributions for the completion ages defined above and, most importantly for our purposes, the *difference* between these ages. The data’s degree of support for our hypothesis that intra-ethnic sharing norms are acquired earlier than inter-ethnic sharing norms can be quantified as the posterior probability that the inter-ethnic completion age is older than the intra-ethnic completion age, i.e., the posterior probability that the former value minus the latter is greater than zero. Note that this value may be high even if the individual 95% Highest Posterior Density Intervals (HPDI) for the two different completion ages overlap. As outlined in our Registered Report Protocol [94], we interpret a posterior probability exceeding 0.75 as support for our hypothesis. Regardless of the posterior probability obtained, we report posterior mean and 89% HPDI for the completion ages corresponding to all cultures and norms. Note that, in our power calculations (described in [94]) with a sample size of 200 participants, differences in acquisition completion times less than one year were unlikely to be reliably detected regardless of how distinct the sharing probabilities are, while differences greater than four years could be detected even if the developmental shifts in rates of sharing are relatively subtle.

## Results

### Interview results

According to the adults who participated in our group interviews, relationships between BaYaka and Bandongo were historically built upon a foundation of sharing and mutual help. In the past, BaYaka lived in the forest full time, yet would visit their fictive kin in the village to bring them meat and honey. In return, BaYaka usually received manioc, plantains, cooking pots, or metal tools. Bandongo, too, would visit BaYaka in the forest to share resources, like fish, and to participate in subsistence activities together, like hunting. BaYaka-Bandongo relationships were characterized by participants as family relations, lineage relations, clan relations, or more rarely, master-servant relations. Sharing occurred within the context of specific BaYaka and Bandongo kin ties:

“…each BaYaka clan line belongs to a respective Bandongo family. As such, the sharing was done according to the alliances.”—Bandongo woman

Economic specialization was a key driver for sharing, as one Bandongo man summarized:

“…because our BaYaka brothers don’t practice on the river, but in the forest. So, when our grandparents went fishing, they shared fish with BaYaka because they [BaYaka] shared also meat with them in return.”

BaYaka report that their ancestors primarily shared with Bandongo for love (*bolingo)* or for joy (*esengo*), as they would with any friend or family member. Bandongo, for their part, recognize BaYaka as the traditional owners of the forest. Sharing is a way in which Bandongo reciprocate BaYaka for their help in orienting their ancestors to the land:

“…the village in which we live was discovered by a Mwaka [BaYaka person], the ponds we own also, they [BaYaka] are in reality the owners. Indeed, seeing how they are real compasses, we made it thanks to them, we were obliged to host them and share with them.”—Bandongo man

In our group interviews with children and adolescents, similar cultural logics are evident. A BaYaka girl stated she shared with Bandongo:

“…because we love each other, we’re family.”

Whereas a Bandongo boy justified sharing based on reciprocity:

“…because they could help me one day.”

While BaYaka and Bandongo lived separately in the past, through forced and voluntary relocation, most BaYaka came to settle—at least some of the time—in the villages. Both BaYaka and Bandongo acknowledge that this co-habitation has led to changing sharing patterns. Sharing has become more transactional, based on needs for specific goods, and on services rendered. Bandongo are reliant on BaYaka labour for garden and forest work. For BaYaka, obtaining cigarettes, money, children’s clothes, salt, and soap, are main motivators for sharing with Bandongo. As one BaYaka woman explained:

“You get honey, you give it to a Milo [Bandongo person], Milo sells, he gets things, he gives to you.”

BaYaka and Bandongo collectively report that several factors have exacerbated these changes. The introduction of money has loosened fictive kinship relations and mutual reliance. Relatedly, bushmeat has become an important export to nearby logging towns: as a result, BaYaka have transitioned from sharing excess meat with their Bandongo kin, to hunting on behalf of Bandongo gun owners who sell the meat onwards on the regional market. Because they now live in close proximity, the taboo which prohibited inter-ethnic marriage has broken down, leading to jealousy and rivalry between BaYaka and Bandongo women. These tensions have sometimes led to threats and violence, resulting in some BaYaka pulling away from their kin, and others moving to different villages:

“They prefer [to live with] those who are good to them.”—Bandongo man

Conflict resolution has also shifted. In the past, when BaYaka lived in the forest and Bandongo in the village, some participants report that there were few conflicts surrounding sharing. If BaYaka took on debt they could not repay, their Bandongo kin would intervene on their behalf, and repay the debt in exchange for labour. Both BaYaka and Bandongo acknowledge that debt is a source of stress. BaYaka often take on debt by claiming payment or gifts for forest resources or labour they plan to complete at a later date, such as asking for children’s clothes or a machete in exchange for honey. If these debts are not quickly repaid, additional resources (e.g., honey, palm oil, fish, vines, black pepper) or labour may be expected. If a debt remains unpaid by either party, the village chief would be consulted, and sanctions may be imposed on the offending party.

BaYaka and Bandongo both report that sharing in the forest occurs differently than sharing in the village. Specifically, in the forest BaYaka must:

“…share with each person until it [the food] is done”—BaYaka man

Whereas, in the village, sharing is more restricted, and everyone eats in their own place. Still, an ethos of generalized sharing not only persists, but is often extended to Bandongo kin:

“You share with everyone. I give my family a bit, then I go to share with my Milo a bit.”—BaYaka woman

For Bandongo, where most time spent with BaYaka in the forest is in the context of hunting or fishing trips, sharing has to be carefully balanced. In recognition that BaYaka perform indispensable labour and guarantee survival in the forest, and for fear of being abandoned if they offend, Bandongo share extensively. Yet, Bandongo also acknowledge that the resources they provide to BaYaka (e.g., cigarettes, manioc) are finite in the forest, whereas sharing is easier in the village, where resources can be more easily replenished. Overall, however, Bandongo continue to share with BaYaka, because:

“…without them we are nothing. That’s why we have to share with them to consolidate our relations”—Bandongo man

In our qualitative interviews, BaYaka and Bandongo adults report that both intra- and inter-ethnic sharing is learned primarily from parents, and that this has not changed over time. Similarly, BaYaka and Bandongo children and adolescents overwhelmingly report learning intra- and inter-ethnic sharing from their parents. These same trends emerged in the free-listing: Table 2 shows frequency and Smith’s Salience for free-lists with adults, regarding from whom intra-and inter-ethnic sharing is learned. For intra-ethnic sharing, BaYaka reported learning from a mean of 2.74 individuals (Max = 5), whereas Bandongo reported learning from a mean of 3.11 individuals (Max = 6). For inter-ethnic sharing, BaYaka reported learning from a mean of 2.35 individuals (Max = 4), and Bandongo reported learning from a mean of 1.80 individuals (Max = 5). For both Bandongo and BaYaka, mothers and fathers were salient transmitters of intra- and inter-ethnic sharing knowledge. Further, the salience of mothers and fathers was relatively high for intra-ethnic sharing; in comparison, salience was more distributed for inter-ethnic sharing.

**Table 2:** Frequency and Smith’s Salience for intra- and inter-ethnic sharing by ethnicity. First five most salient categories per community and sharing norm in bold.

	Bandongo				BaYaka			
	*Intra-Ethnic Sharing*		*Inter-Ethnic Sharing*		*Intra-Ethnic Sharing*		*Inter-Ethnic Sharing*	
	Frequency	Smith’s S	Frequency	Smith’s S	Frequency	Smith’s S	Frequency	Smith’s S
**Mother**	**26**	**0.626**	**13**	**0.307**	**26**	**0.769**	**11**	**0.304**
**Father**	**23**	**0.502**	**11**	**0.251**	**15**	**0.378**	**5**	**0.160**
**Self**	**7**	**0.138**	**11**	**0.281**	**6**	**0.177**	0	0.000
**Peer**	**9**	**0.127**	**4**	**0.065**	**11**	**0.168**	**9**	**0.173**
**Aunt**	**6**	**0.100**	3	0.050	0	0	0	0.000
**Both Parents**	3	0.086	2	0.043	0	0	0	0.000
**In-Law**	3	0.067	2	0.029	0	0	0	0.000
**Brother**	6	0.063	0	0.000	2	0.044	1	0.038
**Spouse**	3	0.051	2	0.048	0	0	2	0.038
**Grandmother**	3	0.043	**7**	**0.133**	3	0.051	1	0.038
**Sister**	4	0.034	1	0.021	4	0.049	2	0.051
**School**	3	0.034	0	0.000	1	0.013	3	0.029
**Family**	2	0.027	0	0.000	**11**	**0.161**	**13**	**0.304**
**Uncle**	1	0.007	1	0.014	1	0.011	2	0.064
**Work**	1	0.007	1	0.029	0	0	0	0.000
**Bandongo**	0	0.000	0	0.000	3	0.024	**9**	**0.346**
**Grandfather**	0	0.000	3	0.057	1	0.008	1	0.010

*N.B.—As the data are based on free responses, some categories contain overlap.*

Peers were in the top five most salient transmitters for intra- and inter-ethnic sharing in both communities. As one BaYaka woman stated:

“[I learned] because I was given things and I had to give things back to those I played with.”

Bandongo listed aunts as important transmitted of intra-ethnic sharing, and grandmothers for inter-ethnic sharing. A Bandongo man said:

“My grandmother would always tell me that once in the village you must share with BaYaka to gain their trust.”

Bandongo also reported that they learned both intra- and inter-ethnic sharing by themselves. A Bandongo woman explained:

“I told myself, if a Mwaka asks me I have to give him something, because he is like me.”

BaYaka reported learning intra-ethnic sharing by themselves; we note, however, that in follow-up questions, many such instances in fact appear to relate to observation, as one BaYaka man elaborates:

“I learned to share myself, by looking at other people, it’s what pushed me to share.”

Both intra- and inter-ethnic sharing norms were learned by BaYaka from their extended family. For inter-ethnic sharing, Bandongo were also named as knowledge transmitters. As one BaYaka woman explained:

“I was given things like pots, spoons, plates, and I also gave to her, because she helped me with many things. When you find a Bandongo friend who shares things really you are well in the village.”

Table 3 shows the frequency and percent of adult participants who reported learning via one of the four mechanisms observed in our dataset. Some cross-cultural differences are apparent. Specifically, for intra-ethnic sharing, more Bandongo reported learning via Instruction than BaYaka. A Bandongo woman explained:

**Table 3:** Frequency (%) of teaching and learning types (N_Bandongo_ = 35, N_BaYaka_ = 31).

	Bandongo		BaYaka	
Type	*Intra-Ethnic*	*Inter-Ethnic*	*Intra-Ethnic*	*Inter-Ethnic*
**Instruction**	22 (62.86)	15 (42.86)	4 (12.90)	3 (9.68)
**Observation/Imitation**	8 (22.86)	12 (34.29)	24 (77.42)	22 (70.97)
**Play**	0	0	1 (3.23)	0
**Self**	5 (14.29)	8 (22.86)	2 (6.45)	1 (3.23)
**No Answer**	0	0	0	5 (16.13)

“One time, my dad found me cooking, and when I finished, I ate everything by myself. My father pointed out through my behaviour that I shouldn’t just keep things to myself, but also give to others, even if it’s just a little. After that, I realized myself that if I don’t share, no one will share with me.”

In contrast, more BaYaka reported learning intra-ethnic sharing via Observation/Imitation than Bandongo. A BaYaka man said:

“I looked at older children and how older children shared, and returned by sharing with friends.”

For inter-ethnic sharing, BaYaka continued to report Observation/Imitation as the main mode of transmission. A BaYaka woman said:

“When she [a Bandongo woman] cooked food like *jabuka* she gave me some food, and after when I saw how she started to give me things, me too I had to give her things because it did me good.”

For inter-ethnic sharing, Bandongo learning via Instruction and Observation/Imitation was more evenly distributed. One Bandongo man said:

“Dad lived more with the BaYaka, I saw how he treated them, and he would say: ‘you have to share with them because they will help you.’”

These trends are also echoed in our group interviews with BaYaka and Bandongo children and adolescents; BaYaka girls and boys overwhelmingly reported learning to share within and across ethnicity through observation, such as by watching their mothers distribute plates of food, and watching their fathers collect and distribute honey. Bandongo girls and boys reported learning to share within and across ethnicity by observing their parents share resources like meat and fish, by being taught the virtues of sharing, or being taught the history of Bandongo relationships with BaYaka.

**Fig 2:**
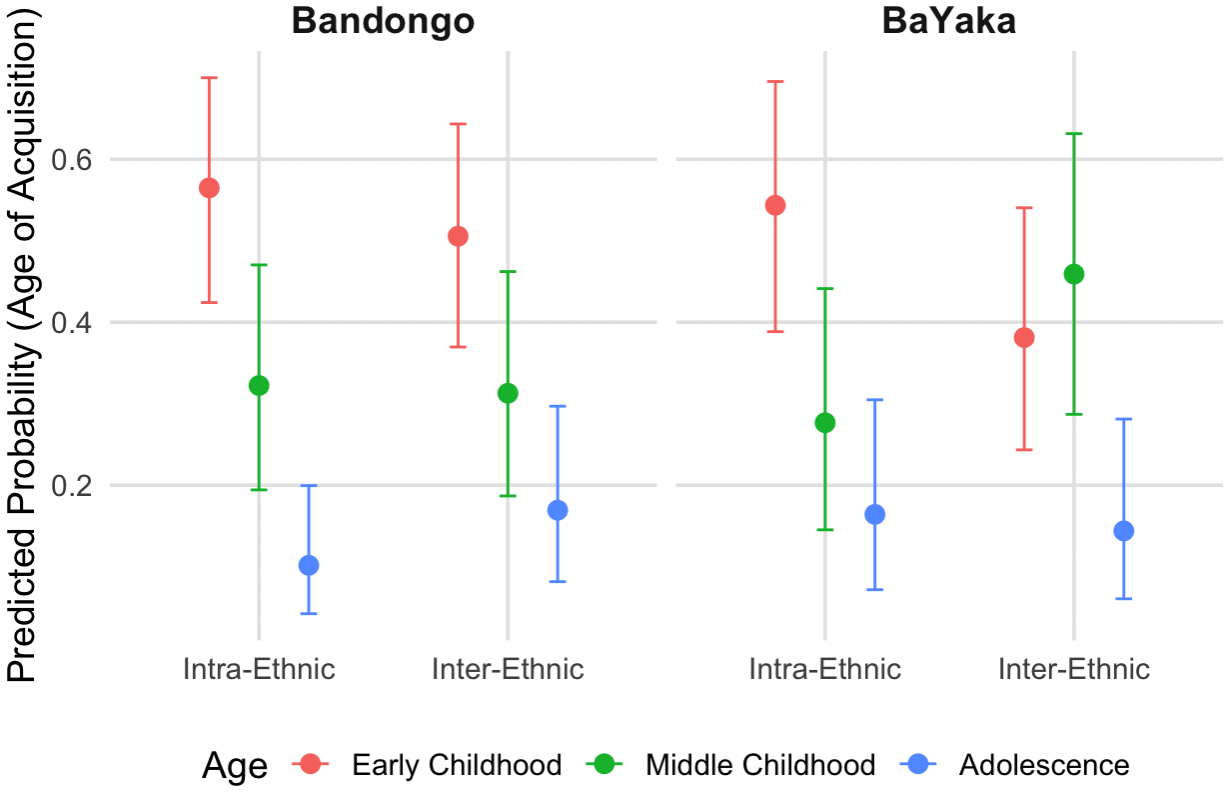
Adults’ reported age of knowledge acquisition. Multinomial regression results regarding adult (N = 66) reports regarding age of sharing knowledge acquisition, with 89% Credible Intervals.

Results for the model analyzing adult reports regarding age of sharing knowledge acquisition are visualized in Fig 2 (see also S2 and S3 Tables). Across the board, Bandongo adults reported primarily having learned intra- and inter-ethnic sharing in early childhood. BaYaka adults reported having learned intra-ethnic sharing in early childhood, whereas BaYaka reports of learning inter-ethnic sharing in early and middle childhood are comparable.

**Fig 3:**
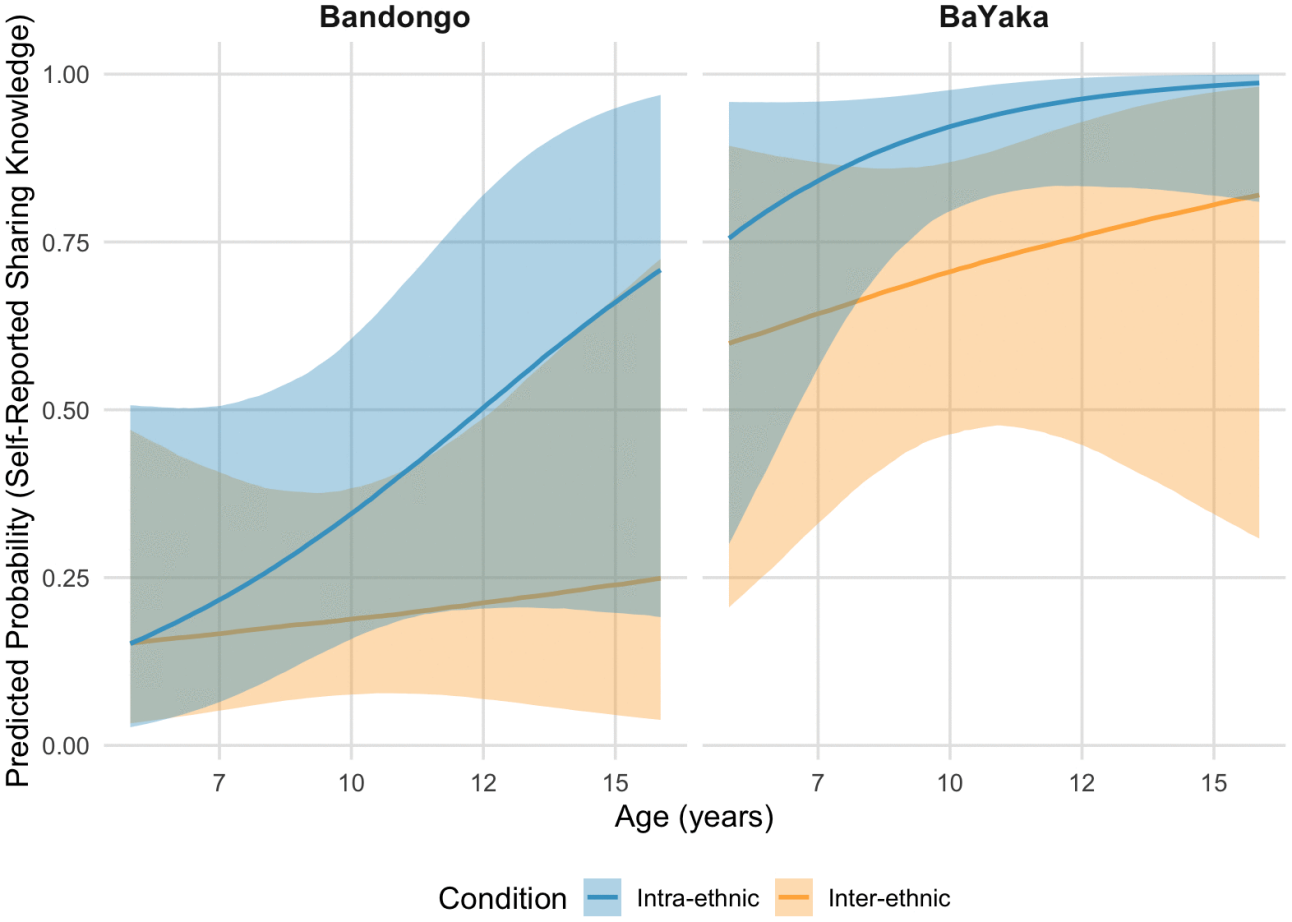
Children’s self-reported sharing knowledge. Logistic regression results regarding children’s (N = 115) post-interview yes/no responses to whether they know intra- and inter-ethnic sharing, with 89% Credible Intervals.

Results for the model analyzing children’s post-interview yes/no responses to whether they know intra- and inter-ethnic sharing are visualized in Fig 3 (see also S4 and S5 Tables). These results suggest that a greater proportion of BaYaka than Bandongo children reported knowing how to share. In both groups, more children reported knowing how to share within ethnicity than across, and this difference increases with age. Further, Fig 3 suggests that this age-related change is driven more by developmental increases in children’s self-reported intra-ethnic sharing knowledge than their self-reported inter-ethnic sharing knowledge.

### Experimental results

The ‘infant’ sharing rate, which is not specific to either culture or condition, was estimated to be 0.42, 89% HPDI [0.32, 0.52]. We estimated four distinct adult sharing rates, one per combination of culture and condition (marginalizing over gender effects): Bandongo intra-ethnic sharing = 0.45, 89% HPDI [0.29, 0.61]; Bandongo inter-ethnic sharing = 0.36, 89% HPDI [0.23, 0.50]; BaYaka intra-ethnic sharing = 0.40, 89% HPDI [0.26, 0.54]; BaYaka inter-ethnic sharing = 0.40, 89% HPDI [0.26, 0.56].

**Fig 4:**
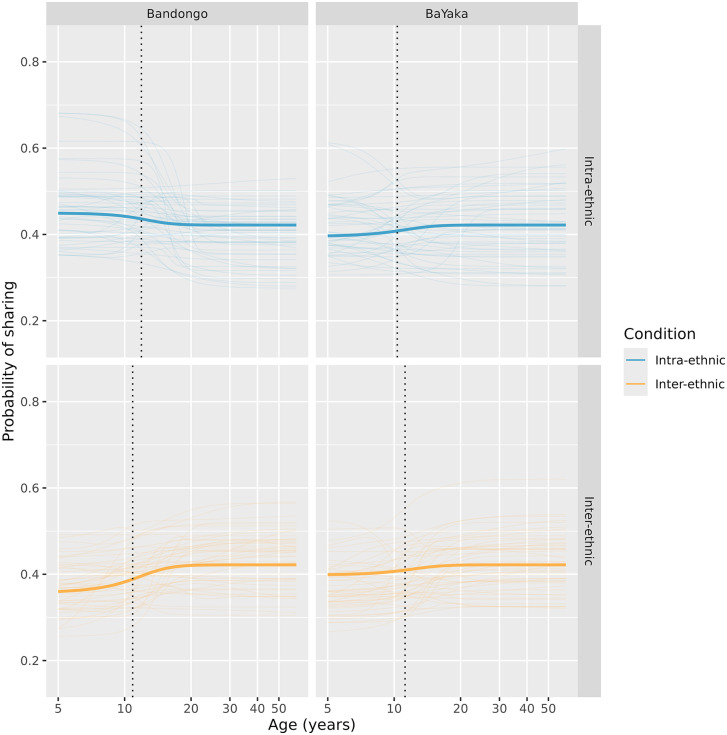
Developmental trajectories for sharing. Posterior predictive plots, marginalizing out gender effects, showing the probability of choosing to share in Inter- and Intra-ethnic conditions in the Dictator Game across development (N = 179). Thick lines show posterior mean predictions; thin lines show individual posterior samples. The dotted vertical line is the completion age, i.e., the age when 89% of the change between the infant and adult sharing probabilities has taken place.

We did not find support for our hypothesis that intra-ethnic sharing norms are acquired earlier than inter-ethnic sharing norms. Specifically, Bandongo participants’ mean completion age for intra-ethnic sharing was 13.31 years, 89% HPDI [6.15, 20.23], and for inter-ethnic sharing was 12.15 years, 89% HPDI [6.05, 18.43]. BaYaka completion age for intra-ethnic sharing was 11.51 years, 89% HPDI [5.12, 18.29], and for inter-ethnic sharing was 12.46 years, 89% HPDI [6.02, 19.19]. Contrasts between intra- and inter-ethnic sharing by ethnicity can be found in Table 4. These show small and uncertain differences in completion ages, with posterior probabilities of positive contrasts in our hypothesized direction very close to 0.5. Fig 4 further demonstrates that for both BaYaka and Bandongo, completion ages were similar in intra and inter-ethnic sharing conditions.

**Table 4:** Contrasts estimating the difference in completion ages for the development of Bandongo and BaYaka children’s intra- vs inter-ethnic sharing choices.

	Completion age difference (inter – intra) posterior mean	Completion age difference 89% HPDI	Posterior probability of predicted positive difference
**Bandongo**	−1.16	[-10.72, 8.1]	0.43
**BaYaka**	0.95	[-8.31, 10.0]	0.57

## Discussion

In this paper, we investigated the social learning and developmental trajectories of intra- and inter-ethnic sharing norms in a multi-ethnic community in the Republic of the Congo. Drawing on qualitative and quantitative interviews and a modified Dictator Game, we aimed to understand how, when, and from whom children acquire the norms that regulate inter-group cooperation. Based on our previous ethnographic research, we predicted that BaYaka and Bandongo children would acquire intra-ethnic sharing norms earlier than inter-ethnic sharing norms. However, our findings did not support this prediction. Instead, we found that both types of norms developed along similar timelines, with norm acquisition occurring primarily by middle childhood for both BaYaka and Bandongo participants. This result aligns with prior research showing that children across cultures become increasingly sensitive to and conform to social norms during middle childhood [19,29,35]. These findings suggest that out-group cooperation norms do not necessarily require more time, teaching, or cognitive effort to learn. Instead, when social contexts regularly expose children to out-group collaboration, such as through shared labour, fictive kinship, and inter-household exchanges, inter-ethnic norms may be learned as part of a broader cooperative repertoire.

Fig 3 further suggested that the overall proportion of BaYaka children who reported knowing how to share, both within and across ethnicity, was higher than that of Bandongo children. This cross-cultural difference may reflect society-specific sharing norms. As noted in the present study and in the published literature, BaYaka sharing norms are generalized, egalitarian, and structured around direct and visible practices such as demand sharing and collective consumption [79,83,82]. In contrast, Bandongo sharing norms are often structured around status, kin obligation, and reciprocal expectations, and thus may require children to manage more complex social information in order to apply them. This difference in norm complexity may help explain why Bandongo children, particularly in the inter-ethnic condition, were less likely to report knowing how to share.

Differences in norm complexity may also explain our findings regarding learning mechanisms. Bandongo participants were more likely to report learning intra-ethnic sharing via instruction than BaYaka participants. In contrast, BaYaka reported learning both intra- and inter-ethnic norms predominantly through observation and imitation. As direct instruction is often used to transmit complex knowledge [102–104], the greater reliance on teaching among Bandongo participants may be tied to the conditionality of their sharing norms. Differences in learning mechanisms may also reflect social ecologies: Compared to BaYaka [105], Bandongo households have fewer inhabitants, more private spaces, and greater distances between neighbouring households. BaYaka thus have more opportunities to observe multiple instances of sharing from their own households and from neighbours, potentially leading to the development of a generalized sharing rule without the need for much explicit instruction.

We also examined *from whom* norms are acquired. Previous research suggests that parents play a prominent role in the transmission of sharing knowledge [59], and in shaping beliefs about in-group and out-group members [71]. Similarly, in the present study parents were relatively salient transmitters of intra- and inter-ethnic sharing norms for both BaYaka and Bandongo. The prominence of peers in terms of norms socialization—observed in other studies [64,106,107]—was also evidenced in free-list responses. Further, sharing knowledge was transmitted by multiple sources. This ‘many-to-one’ transmission “ensures not only high conservation but also high uniformity” across individuals [108]. In turn, uniformity promotes the coordination needed for sharing norms to be successful [8]. Interestingly, we found that mothers and fathers had relatively high salience for intra-ethnic sharing, meaning that they were named at the top of nearly all participant free-lists. While mothers and fathers also played an important role in the acquisition of inter-ethnic sharing norms, their salience was relatively lower because fewer participants named them during the free-listing task. Instead, salience was more distributed across a wider range of knowledge transmitters. For Bandongo, this included grandmothers, whereas for BaYaka, this included one’s extended family, and Bandongo individuals. That inter-ethnic sharing norm acquisition primarily occurred from within one’s extended household—which can include out-group fictive kin [109]—echoes our group interviews with adults, where participants reported that inter-ethnic sharing historically occurred between specific allied lineages (see also [85]). Together, these data suggest that inter-ethnic sharing norms are negotiated between specific families, rather than standardized across the community as a whole.

### Limitations and future directions

Our study has several limitations. First and foremost, our final sample size for the Dictator Game was smaller than intended, and the distribution of participants across age groups was uneven (S1 Fig). This limited our ability to capture small effects. Moreover, our reliance on retrospective self-reports may underrepresent forms of learning that are indirect or less easily verbalized. Indeed, instruction is a highly conspicuous form of teaching [110]. Yet, while no BaYaka participants reported learning sharing via task assignment, during our fieldwork in forest camps and villages, we have observed children tasked with bringing plates to different households every evening. In another study of Aka children’s learning to share, children explicitly reported being told to watch how the plates are served before they were asked to distribute them [61]. And, as participants in our group interviews report, such plates are sometimes also shared with Bandongo kin. Further, Boyette and Hewlett [111] found that among the Aka, teaching via instruction was reserved for subsistence skills, whereas social norms were transmitted via teasing and negative feedback. These more subtle forms of teaching (task assignment, teasing, negative feedback) are more likely to be embedded into everyday activities, and thus, harder to recall, explaining their infrequent mention in our interviews with adults (see also [112]).

In this study, we intentionally did not compare the relative propensity to give to in- versus out-group members. This is because, as Pisor et al. [113] argue, “the validity of economic games—especially games featuring minimalistic instructions, anonymous recipients and money—is compromised in some populations, such as those that are less market integrated and predominantly interact with known individuals, not with strangers.” As such, whether BaYaka and Bandongo give or not in the Dictator Game is unlikely to reflect actual sharing in much more complex real-world scenarios with known community members. Instead, our study used a modified Dictator Game to interpret similarity in behaviour at different stages of development within this constrained experimental context. More flexible economic games, such as the recipient identity-conditioned heuristics (RICH) games [114], could shed light on how sharing norms are practiced within participants’ real social networks.

More broadly, though this work supports prior empirical findings from cross-cultural research that highlights middle childhood and early adolescence as important periods for children’s sensitivity to (and internalization of) norms for fairness and sharing [37,115,36], it does so by introducing robust research methods that avoid the limitations of prior research. Previous studies have largely explored the development of children’s understanding of sharing by measuring developmental differences in behaviour across diverse societies, with the expectation that these societies are more likely to hold different norms for sharing. However, the selected societies often display other cultural differences (e.g., climate, ecology, wealth, health, educational approaches) which can introduce confounding levels of societal variation in related behaviours. The present study avoids this issue in two ways. First, it studies different societies living in the same local areas, minimizing the sources of confounding cultural variation which come from comparing studies at great distance. Secondly, it explores the development of children’s understanding of multiple specific norms within single societies (e.g., norms for inter-ethnic and intra-ethnic sharing). Future researchers could similarly explore the development of children’s understanding of norms without the complexities of large cross-cultural samples by identifying different sharing norms within individual societies (e.g., norms for sharing with specific recipients in specific contexts) and study how children’s behaviour comes to align with those norms with age. Future research could also investigate the relative salience of different models in the acquisition of intra- and inter-ethnic sharing norms in other cultures, including in WEIRD contexts.

### Conclusion

The relationships between BaYaka and Bandongo studied in the present paper are but one example of cooperation between Congo Basin foragers and farmers regulated by economic and cultural exchange [87,116,117]. Such inter-group cooperation also occurs across the globe [21–24]. We have demonstrated that norms for sharing within and across groups develop by middle childhood through a combination of instruction and observation, and are transmitted by a range of models. These findings contribute to broader debates in anthropology, psychology, and cultural evolution regarding how social norms are acquired and maintained. By focusing on the ontogeny of inter-ethnic sharing, an understudied topic [23], we have shed light on how children come to participate and sustain cooperation in multi-ethnic communities.

## Supporting information

S1 File This file details deviations for the planned analyses, and includes supplementary tables and figures. (PDF)
