# Supplementary material for: The social learning and development of intra- and inter-ethnic sharing norms in the Congo Basin
Source: PLoS One. 2026 Jan 28;21(1):e0340388. doi: 10.1371/journal.pone.0340388 (PMC12851454; doi:10.1371/journal.pone.0340388)
Supplement: S1 File — (PDF) [file pone.0340388.s001.pdf]

Supplementary Materials for The social learning and development of intra- and inter-ethnic sharing norms in the Congo Basin

Deviations

- **Timeline:** We originally planned to collect interview and experimental data in the same field season. However, during piloting of our questions, we realized that our questions were not as easily understood by participants as we had anticipated. We decided to postpone interview data collection to allow us to reformulate the questions. As a result, the experiment and interviews were not always conducted with the same participants.
- **Experimental sample size:** We originally intended to have a sample size of 200 participants. However, after exclusions due to comprehension questions and experimenter error, our final sample size was 179 participants. Further, because many adolescents were not in the village during data collection due to schooling or subsistence activities, the distribution of participants across age groups was not even (Figure S1). As a result, it is unlikely that we would have been able to capture small effects.
- **Questions:** During the interview we asked participants about the context in which they learned to share (e.g., where were you, what were you doing). This question was variably understood by participants, making interpretation difficult. We thus opted to exclude this question for our results.
- **Social learning codes (Table 1):** We added the category of 'self' to the social learning coding, as many participants responded that they had learned to share themselves.
- **Experimental materials:** While we originally planned to use beads as the reward, during piloting we noted that the difference between keeping one and two beads was not salient enough for participants. We thus opted to use a locally available small candy as the reward instead, which we found to be more motivating for participants of all ages.
- **Interview analysis:** We originally planned to conduct a series of Fisher's exact tests. After reviewing our data, we opted to use different methods (salience analysis, frequencies, multilevel models) because we felt they better represented the data. These changes were made prior to data analysis. None of these tests were intended to be inferential.
- **Experimental modelling.** The distribution of girls/women and boys/men experimental participants was uneven. We thus adjusted for participant gender in the models.

S1 Table. Focus group composition and length.

No.	Gender	Age	Ethnicity	Village	Length (min.s)
1	Men	Adult	Bandongo	Village 1	33.32
2	Men	Adult	Bandongo	Village 1	16.08
3	Women	Adult	Bandongo	Village 1	9.39
4	Women	Adult	Bandongo	Village 1	16.12
5	Men	Adult	Bandongo	Village 2	25.54
6	Men	Adult	Bandongo	Village 2	26.38
7	Women	Adult	Bandongo	Village 2	18.42
8	Men	Adult	BaYaka	Village 1	12.55
9	Men	Adult	BaYaka	Village 1	17.17
10	Men	Adult	BaYaka	Village 1	18.56
11	Women	Adult	BaYaka	Village 1	34.57
12	Women	Adult	BaYaka	Village 1	13.46
13	Women	Adult	BaYaka	Village 2	17.07
14	Boys	Child	Bandongo	Village 1	2.52
15	Boys	Child	Bandongo	Village 1	6.55
16	Girls	Child	Bandongo	Village 1	4.48
17	Boys	Child	Bandongo	Village 2	4.57
18	Boys	Child	Bandongo	Village 2	3.14
19	Boys	Child	Bandongo	Village 2	4.33
20	Girls	Child	Bandongo	Village 2	6.33
21	Girls	Child	Bandongo	Village 2	4.15
22	Boys	Child	BaYaka	Village 1	8.28
23	Boys	Child	BaYaka	Village 1	4.57
24	Girls	Child	BaYaka	Village 1	5.34
25	Girls	Child	BaYaka	Village 1	4.00

S2 Table. Number of adults ($N_{Bandongo}$=35, N_{BaYaka}=31) reporting the age at which they learned intra- and inter-ethnic sharing.

	**Bandongo**		**BaYaka**	
Age	Intra-Ethnic	Inter-Ethnic	Intra-Ethnic	Inter-Ethnic
Early Childhood	20	17	17	9
Middle Childhood	12	9	8	13
Adolescence	3	7	6	4

N.B. two Bandongo adults reported they learned inter-ethnic sharing as adults. Five BaYaka adults reported that they never learned inter-ethnic sharing.

S3 Table. Parameter Estimates and 95% Credible Intervals (CI) for multinomial model analyzing adult (N=66) reports regarding the age category in which they learned to share. Values in bold are those for whom CIs do not cross zero.

Parameter	Median	CI_low	CI_high
b_muMiddle_Intercept	-0.484	-1.374	0.311
b_muAdo_Intercept	**-1.087**	**-2.171**	**-0.174**
b_muMiddle_GenderM	-0.102	-0.956	0.760
b_muMiddle_conditionintra	-0.079	-0.965	0.803
b_muMiddle_EthnicityBaYaka	0.664	-0.304	1.660
b_muMiddle_conditionintra:EthnicityBaYaka	-0.787	-1.972	0.380
b_muAdo_GenderM	0.051	-0.936	1.021
b_muAdo_conditionintra	-0.616	-1.706	0.407
b_muAdo_EthnicityBaYaka	0.114	-0.979	1.217
b_muAdo_conditionintra:EthnicityBaYaka	0.403	-0.905	1.721

S4 Table. Number of children (N=155) per age group (%) reporting knowing intra- and inter-ethnic sharing.

	**Bandongo**		**BaYaka**	
Age	Intra-Ethnic	Inter-Ethnic	Intra-Ethnic	Inter-Ethnic
Early Childhood	6 (42.86)	5 (35.71)	14 (77.78)	14 (77.78)
Middle Childhood	12 (40.00)	9 (28.12)	25 (83.33)	19 (63.33)
Adolescence	3 (37.50)	2 (22.22)	11 (91.67)	10 (83.33)

N.B. Two Bandongo children in Middle Childhood and one Bandongo Adolescent declined to respond to, or did not know the answer to, the intra-ethnic sharing question. Early childhood: 5-7 years, Middle childhood: 8-12 years, Adolescence: 13-16 years.

S5 Table. Parameter Estimates and 95% Credible Intervals (CI) for logistic model analyzing children's (N=115) post-interview yes/no responses regarding whether they know intra- and inter-ethnic sharing. Values in bold are those for whom CIs do not cross zero.

Parameter	Median	CI_low	CI_high
b_Intercept	**-1.452**	**-2.726**	**-0.233**
b_genderM	0.560	-0.671	1.821
b_conditionintra	0.824	-0.145	1.838
b_age_z	0.146	-0.802	1.120
b_EthnicityBaYaka	**2.338**	**1.102**	**3.617**
b_conditionintra:age_z	0.514	-0.437	1.508
b_conditionintra:EthnicityBaYaka	0.781	-0.431	2.073
b_age_z:EthnicityBaYaka	0.131	-1.094	1.377
b_conditionintra:age_z:EthnicityBaYaka	-0.006	-1.309	1.299

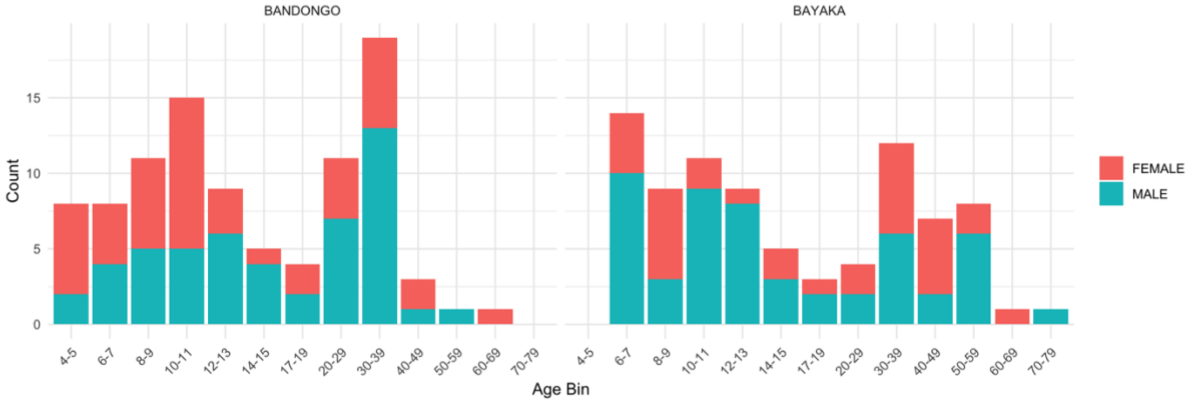

S1 Figure. Demographic overview of Dictator Game participants.
